# Drivers of the taxonomic and functional structuring of aquatic and terrestrial floodplain bird communities

**DOI:** 10.1007/s10980-024-01948-3

**Published:** 2024-09-04

**Authors:** Gábor Ónodi, István Czeglédi, Tibor Erős

**Affiliations:** 1 HUN-REN Balaton Limnological Research Institute, Klebelsberg Kuno Utca 3., Tihany, 8237 Hungary; 2 National Laboratory for Water Science and Water Security, HUN-REN Balaton Limnological Research Institute, Klebelsberg Kuno Utca 3., Tihany, 8237 Hungary

**Keywords:** Functional traits, Land cover, Habitat structure, Spatial factors, Variance partitioning

## Abstract

**Context:**

There has been a limited amount of research which comparatively examines the local and landscape scale ecological determinants of the community structure of both riparian and aquatic bird communities in floodplain ecosystems.

**Objectives:**

Here, we quantified the contribution of local habitat structure, land cover and spatial configuration of the sampling sites to the taxonomical and functional structuring of aquatic and terrestrial bird communities in a relatively intact floodplain of the river Danube, Hungary.

**Methods:**

We used the relative abundance of species and foraging guilds as response variables in partial redundancy analyses to determine the relative importance of each variable group.

**Results:**

Local-scale characteristics of the water bodies proved to be less influential than land cover and spatial variables both for aquatic and terrestrial birds and both for taxonomic and foraging guild structures. Purely spatial variables were important determinants, besides purely environmental and the shared proportion of variation explained by environmental and spatial variables. The predictability of community structuring generally increased towards the lowest land cover measurement scales (i.e., 500, 250 or 125 m radius buffers). Different land cover types contributed at each scale, and their importance depended on aquatic vs terrestrial communities.

**Conclusions:**

These results indicate the relatively strong response of floodplain bird communities to land cover and spatial configuration. They also suggest that dispersal dynamics and mass-effect mechanisms are critically important for understanding the structuring of floodplain bird communities, and should therefore be considered by conservation management strategies.

**Supplementary Information:**

The online version contains supplementary material available at 10.1007/s10980-024-01948-3.

## Introduction

Floodplains are essential components of natural riverine landscapes, which maintain high biodiversity due to the heterogeneity in the structure and spatial configuration of terrestrial and aquatic habitats (Ward et al. 1999; Thorp et al. 2006). Despite their crucial importance for biodiversity, floodplains are among the most endangered ecosystems globally, caused by large-scale river regulation works, that altered the spatial and temporal complexity of terrestrial, riparian, wetland and open water habitats (Ward et al. 1999; Tockner and Stanford 2002; Habersack et al. 2016). In Europe, which is the most human-dominated continent, up to 90% of former floodplains have been degraded to functional extinction (Tockner et al. 2010), with the degradation of natural hydrological dynamics and ecological processes between the land–water interface. Therefore, a detailed understanding of how both the terrestrial and aquatic environment shapes the structuring of biotic communities in still relatively intact floodplains could provide useful implications for the restoration and conservation management of floodplain ecosystems, especially in temperate regions, where most large rivers lost their floodplains (Hayes et al. 2018; Havrdová et al. 2023).

Birds are important components of the biota of floodplain ecosystems, especially since they occupy both terrestrial and aquatic habitats (Davis 1994; Kingsford et al. 2004; Lorenzón, et al. 2016a, 2019). As birds are conspicuous and mobile vertebrates, they can respond quickly to the dynamic changes of landscapes, which makes them advantageous model organisms in landscape ecological research (Sullivan et al. 2007; Gao et al. 2021). For example, among aquatic birds, different species use the mosaic of dynamically changing floodplains in various ways according to their local environmental characteristics (Kingsford et al. 2004; Lorenzón et al. 2016a). Hydrological dynamics can filter their community composition since there are species groups that prefer more secluded wetlands, silt plateaus, deeper water bodies or even running rivers (Boulton et al. 2008).

Besides the local environment, the spatial configuration of landscape patches can also affect a wide variety of ecological processes, which determine the community structure of birds (Wiens 2002; Thornton et al. 2011; Pérez-García et al. 2014). For example, some forest bird species, such as cavity-nesting species were found to be more sensitive to the surrounding land cover than to the local characteristics of habitat patches (Estades and Temple 1999; Vergara and Armesto 2009; Pérez-García et al. 2014). At the community level, local species richness can also depend on both local and regional landscape-level factors (Ekroos and Kuussaari 2012; Pérez-García et al. 2014). Therefore, a better understanding of the role of local, landscape-level and spatial variables in the structuring of floodplain bird communities could help the conservation of this important vertebrate group. However, despite the recognition of the importance of bird communities in floodplain habitats (Selwood et al. 2017; Lorenzón et al. 2019; Machar et al. 2022), there is currently insufficient knowledge on the structuring of bird communities to both spatial context and environmental characteristics of both terrestrial and aquatic components of the floodplains of large alluvial rivers (Arruda Almeida et al. 2016; Lorenzón et al. 2016b; Fluck et al. 2020).

Characterising trait-environment relationships has been emphasized to be a useful alternative approach for understanding the responses of ecological communities to the heterogeneity of the environment (Erős et al. 2009; Arruda Almeida et al. 2018; Rault et al. 2023). In this regard, functional traits, which directly inform the function of species in the environment proved to be especially important (Erős et al. 2009; Tavares et al. 2015; Arruda Almeida et al. 2018). However, taxonomic and functional characterizations of communities have been developed rather independently (Sheldon et al. 2011; Mouillot et al. 2013; Velásquez-Tibatá et al. 2013). For floodplain bird communities, it is generally unknown how, and to what extent taxonomic- and trait-based community structures show congruent patterns along major environmental gradients, and specifically, what is the role of different explanatory variable groups in predicting taxonomic and trait-based structure (but see e.g., Lorenzón et al. 2016b; Andrade et al. 2018; Aguilar et al. 2021).

Therefore, this study aimed to quantify the relative importance of local habitat structure, land cover and space in the variation of taxonomic and functional structure of both terrestrial-riparian (hereafter terrestrial) and aquatic bird communities across a river-floodplain landscape of the river Danube, Hungary. We were especially interested in determine the individual and shared effects of the above-mentioned variable groups to better understand the predictability of bird communities and the role of landscape context on predictability. In addition, we also examined the role of scale in quantifying the importance of land cover variables, since some studies found this characteristic influential (Henckel et al. 2019; Meffert and Dziock 2013).

For terrestrial bird communities, we predicted that land cover will be the most important variable group, which would determine both taxonomic and trait based structure, which have been shown to be influential determinant in other studies as well (Bossenbroek et al. 2005; Meffert and Dziock 2013; Selwood et al. 2017; Henckel et al. 2019). We also predicted that the predictive power of land cover will increase with decreasing measurement scale of land cover variables (i.e., using different radii around the study sites to characterize the contribution of land cover) since terrestrial birds show a strong affinity to land cover variables (Meffert and Dziock 2013; Henckel et al. 2019). On the contrary, for aquatic bird communities, we predicted the overarching importance of local habitat features of the waterbodies (Lorenzón et al. 2016b, 2019), and especially the importance of hydrological connectivity gradient to the main channel, since this variable can largely determine other features of habitat structure (Tockner et al. 1999; Bolland et al. 2012; Reid et al. 2016). Finally, we also predicted that both local habitat features and land cover variables will be more important predictors of bird communities than purely the spatial location of the sampling sites (i.e., space), since dispersal limitation may less influence birds than is the case for other taxa (Sullivan et al. 2007; Gao et al. 2021), at least at the floodplain scale. Rather, spatial location may interact with other features of the habitat, especially in floodplain ecosystems, where the strong influence of lateral connectivity gradients may increase the joint (i.e., shared) importance of spatial and environmental variables in community structuring. Overall, these results would indicate that landscape context is especially important for understanding the structuring of (floodplain) bird communities (Fletcher and Hutto 2008; Yuan et al. 2014; Lorenzón et al. 2016a).

## Material and methods

### Study area and site selection

**Fig. 1:**
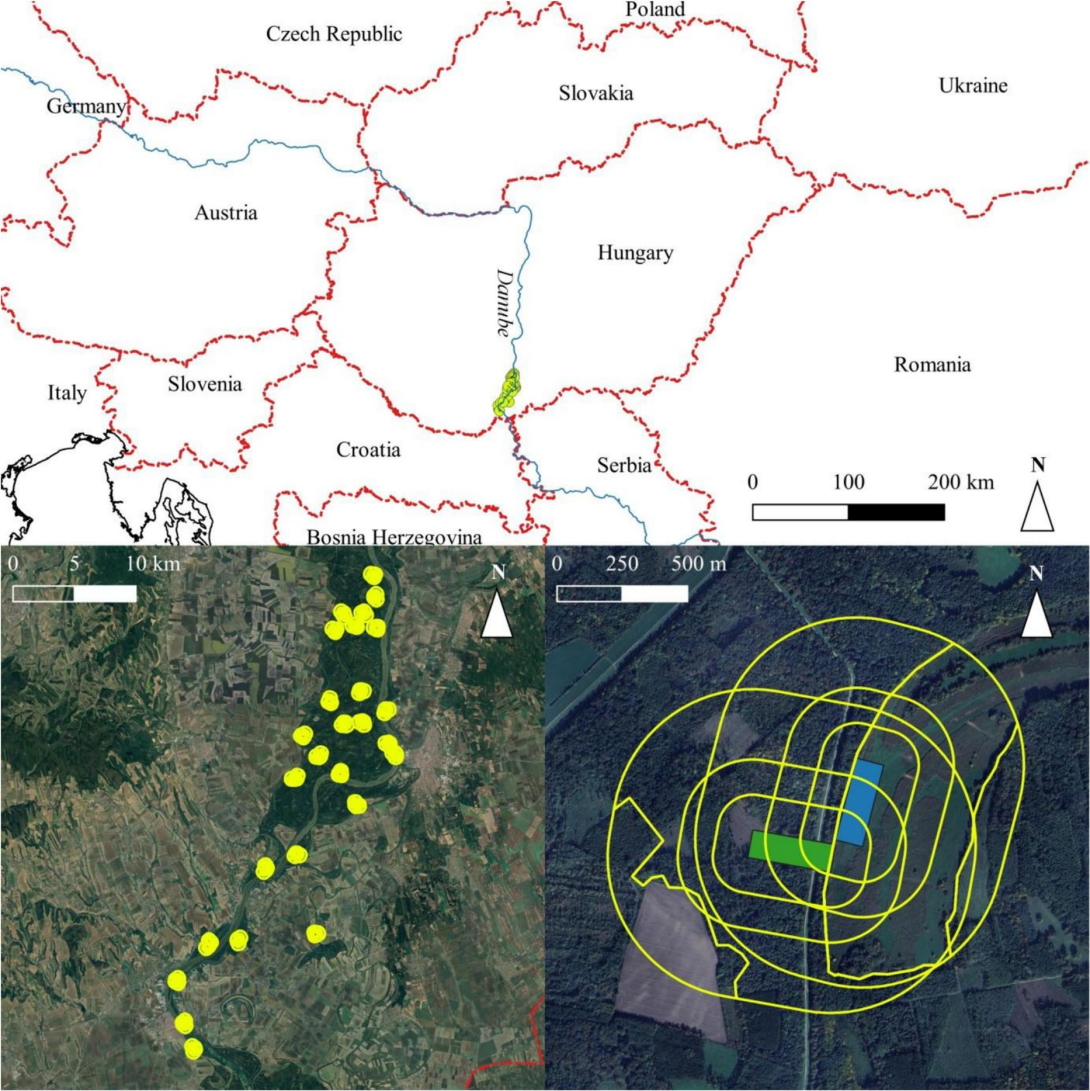
Location of the study area (top), the exact location of the study sites (bottom left) and an example of the aquatic and terrestrial bird transect arrangements with the different buffer zones for land cover measurements (bottom right). In this image the green rectangle indicates a terrestrial bird transect, while the blue one represents an aquatic bird transect

We appointed our study sites in the Middle Danube, Southern Hungary, between river km 1499–1433 (Fig. 1). The river Danube has a mean annual discharge of 2400 m^3^ s^−1^ in this region, with an average slope of about 5 cm km^−1^, and flow velocity of 0.8–1.2 m s^−1^ at mean water level (Schöll and Kiss 2008). This area consists of the largest functioning floodplain in the Middle Danube together with its transboundary Croatian counterpart (Hein et al. 2016; Funk et al. 2019). The major portion of the floodplain is part of the Danube-Dráva National Park and is also included in the list of protected sites in the Ramsar Convention on Wetlands of International Importance Especially as Waterfowl Habitat (Tardy 2007). This area has a variety of floodplain forest habitats (such as willow-poplar and oak-ash-elm floodplain forests), grasslands, agricultural fields, and a diverse array of aquatic habitats like side-arms, backwaters, wetlands, that are connected differently to the mainstream branch of the Danube River (Erős and Bányai 2020; Erős et al. 2023).

A total of 27 waterbodies were selected in the floodplains (Fig. 1) based on three major criteria: (i) to represent a hydrological connectivity gradient from the main river to the most isolated backwaters, (ii) to be located relatively evenly across the study area, (iii) to have no heavy anthropogenic degradation (e.g. oxbows with intense recreational activities were excluded from site selection). The mean distance between the study sites was 14.4 km (min–max range: 1.2–40.8 km).

### Bird census

In 2022, we counted terrestrial and aquatic birds in two separate transects of 100 × 300 m as study plots at each water body. Since bird populations change over time, due to the migrating and nesting phenologies of the species, we mitigated this bias in detection probability by counting the individuals three times from late March to early July (Thompson 2002). There was at least a 1 month time lag between the three field sessions. All birds seen or heard were registered except for flyovers, and we used the maximal abundance of each species of the three visits at each transect in further analyses. To ensure that the maximum number of species was encountered, visits lasted between 1 to 5 h after sunrise (the period of highest bird activity) and were only carried out in suitable weather conditions (low wind, no rain or mist) (Dallimer et al. 2012; Andrade et al. 2018). We visited the sampling sites in alternating orders, to avoid temporal bias in detection probability related to the time of day (Blake 1992; Cornils et al. 2015).

For terrestrial birds, we delineated each rectangular transect perpendicular to the bank of each water body (Fig. 1), starting from the beginning of terrestrial vegetation to represent the horizontal gradient in terrestrial floodplain habitats (modified after Perry et al. 2011; Yabuhara et al. 2019). Transect of aquatic birds was placed parallel with the water body right on the bank (i.e. margin of the waterbody) including the silt plateau, (modified after Sulai et al. 2015; Andrade et al. 2018).The size of all transects was the same (i.e. 100 m wide 300 m long), regardless of the size and shape of the water body.

For the determination of functional structure, both terrestrial (45 species) and aquatic (33 species) birds were assigned to foraging guilds using generally accepted categories (Tables 1 and 2). Aquatic birds were categorized as dabbling ducks, vegetation gleaners, small waders, large waders, divers, fishers and raptors (Tavares et al. 2015; Shuford et al. 2016), while terrestrial birds were categorized as herbivores, ground insectivores, shrub insectivores, bark insectivores, canopy insectivores, omnivores and raptors (Pereira et al. 2014; Czeszczewik et al. 2015).

**Table 1:** List of encountered species and their relative abundances along aquatic bird transects with their assigned foraging guilds

Foraging guild	Common name	Scientific name	Mean relative abundance (%)
Dabbling ducks	Mute swan	*Cygnus olor*	1.78
	Garganey	*Spatula querquedula*	0.55
	Eurasian wigeon	*Mareca penelope*	1.62
	Mallard	*Anas platyrhynchos*	38.41
	Common teal	*Anas crecca*	3.56
	Common pochard	*Aythya ferina*	1.47
	Ferruginous duck	*Aythya nyroca*	0.29
Vegetation gleaners	Common moorhen	*Gallinula chloropus*	0.26
	Common coot	*Fulica atra*	2.19
Small waders	Black-winged stilt	*Himantopus himantopus*	0.14
	Northern lapwing	*Vanellus vanellus*	2.56
	Common ringed plover	*Charadrius hiaticula*	0.99
	Black-tailed godwit	*Limosa limosa*	0.03
	Common sandpiper	*Actitis hypoleucos*	0.49
	Green sandpiper	*Tringa ochropus*	3.12
	Common redshank	*Tringa totanus*	2.02
	Common greenshank	*Tringa nebularia*	0.27
Large waders	Eurasian spoonbill	*Platalea leucorodia*	0.01
	Black-crowned night-heron	*Nycticorax nycticorax*	0.74
	Grey heron	*Ardea cinerea*	10.53
	Purple heron	*Ardea purpurea*	0.12
	Great white egret	*Ardea alba*	2.56
	Little egret	*Egretta garzetta*	8.18
Divers	Great crested grebe	*Podiceps cristatus*	0.42
	Little grebe	*Tachybaptus ruficollis*	0.13
	Pygmy cormorant	*Microcarbo pygmaeus*	0.12
	Great cormorant	*Phalacrocorax carbo*	0.34
Fishers	Black-headed gull	*Larus ridibundus*	0.13
	Common tern	*Sterna hirundo*	0.21
	Common kingfisher	*Alcedo atthis*	1.19
Raptors	Western marsh-harrier	*Circus aeruginosus*	0.25
	Black kite	*Milvus migrans*	2.91
	White-tailed sea-eagle	*Haliaeetus albicilla*	1.31

**Table 2:** List of encountered species and their relative abundances along terrestrial bird transects with their assigned foraging guilds

Foraging guild	Common name	Scientific name	Mean relativeabundance (%)
Herbivores	Common pheasant	*Phasianus colchicus*	0.42
	Common woodpigeon	*Columba palumbus*	2.47
	European turtle-dove	*Streptopelia turtur*	0.33
	Eurasian collared-dove	*Streptopelia decaocto*	0.07
	Common chaffinch	*Fringilla coelebs*	9.24
	Hawfinch	*Coccothraustes coccothraustes*	0.90
	European greenfinch	*Chloris chloris*	0.67
	European goldfinch	*Carduelis carduelis*	0.71
	Yellowhammer	*Emberiza citrinella*	1.32
	Eurasian tree sparrow	*Passer montanus*	0.79
Ground insectivores	Eurasian wryneck	*Jynx torquilla*	0.25
	Eurasian green woodpecker	*Picus viridis*	0.73
	Grey-faced woodpecker	*Picus canus*	0.19
	Northern wren	*Troglodytes troglodytes*	2.11
	Eurasian blackbird	*Turdus merula*	2.50
	Song thrush	*Turdus philomelos*	2.52
	European robin	*Erithacus rubecula*	7.96
	Common nightingale	*Luscinia megarhynchos*	1.13
	White wagtail	*Motacilla alba*	0.63
Shrub insectivores	Black redstart	*Phoenicurus ochruros*	0.21
	Eurasian blackcap	*Sylvia atricapilla*	8.37
	Common chiffchaff	*Phylloscopus collybita*	4.22
Bark insectivores	Great spotted woodpecker	*Dendrocopos major*	4.25
	Syrian woodpecker	*Dendrocopos syriacus*	0.14
	Middle spotted woodpecker	*Leiopicus medius*	1.85
	Lesser spotted woodpecker	*Dryobates minor*	0.58
	Black woodpecker	*Dryocopus martius*	0.67
	Eurasian nuthatch	*Sitta europaea*	4.91
	Short-toed treecreeper	*Certhia brachydactyla*	0.40
	Eurasian treecreeper	*Certhia familiaris*	0.56
Canopy insectivores	Eurasian golden oriole	*Oriolus oriolus*	1.95
	Wood warbler	*Phylloscopus sibilatrix*	0.76
	Marsh tit	*Poecile palustris*	1.93
	Eurasian blue tit	*Cyanistes caeruleus*	3.71
	Great tit	*Parus major*	14.34
	Long-tailed tit	*Aegithalos caudatus*	2.47
	Spotted flycatcher	*Muscicapa striata*	4.51
	Collared flycatcher	*Ficedula albicollis*	2.79
Omnivores	Common cuckoo	*Cuculus canorus*	0.78
	Carrion crow	*Corvus corone*	0.24
	Eurasian jay	*Garrulus glandarius*	0.92
	Common starling	*Sturnus vulgaris*	4.15
Raptors	Eurasian buzzard	*Buteo buteo*	0.14
	Black kite	*Milvus migrans*	0.08
	White-tailed sea-eagle	*Haliaeetus albicilla*	0.12

### Local scale habitat variables

The surface area of each waterbody was measured using a Geographical Information System (QGIS v.3.16; QGIS Development Team 2022) and Google Earth Pro.

Hydrological connectivity was defined as a percent proportion of days in a year a waterbody is connected to the main channel (river Danube) (Reckendorfer et al.; 2006; Funk et al. 2013). Mean depth and mean current velocity were measured using a digital terrain model and a water velocity meter, respectively. Within waterbodies, habitat structure was further characterized using visually estimated percentage composition of the following variables at the place of the samplings: emergent, submerged and floating vegetation, floating algae, open water habitat and woody debris. The bank structure was similarly characterized using the following variables: percentage cover of woody (i.e., tree or large bushes) and herbaceous vegetation, canopy cover and cover of artificial surfaces (concrete, rip-rap). The percentage cover of substratum types was visually estimated using the following categories: silt, sand, gravel, and rock. For the general characteristics of the environmental variables, see Table 3.

**Table 3:** Descriptive statistics of local habitat structure variables and their correlation with the first three principal components of the local habitat variables PCA

	Unit	Mean	SD	Min	Max	PC1 (42.05%)	PC2 (12.70%)	PC3 (12.31%)
Area	ha	62.63	115.47	0.30	464.37	0.79	0.44	− 0.25
Depth	m	1.31	1.25	0.40	6.00	0.87	0.39	− 0.15
Emergent plants	%	9.25	15.64	0.00	75.00	− 0.54	− 0.08	− 0.13
Submerged plants	%	6.48	12.33	0.00	57.50	− 0.5	0.46	− 0.31
Floating plants	%	16.56	23.09	0.00	80.00	− 0.59	0.47	− 0.03
Surface algae	%	0.29	1.44	0.00	7.50	− 0.28	0.34	− 0.47
Open water	%	64.38	33.73	3.00	100.00	0.79	− 0.43	0.21
Woody debris	%	3.04	6.70	0.00	35.00	− 0.56	0.15	− 0.14
Woody/trees	%	66.54	28.95	0.00	100.00	0.16	0.54	0.78
Herbaceous	%	31.88	29.13	0.00	100.00	− 0.24	− 0.53	− 0.76
Artificial	%	1.58	3.02	0.00	10.00	0.77	− 0.07	− 0.18
Canopy cover	%	21.71	25.84	0.00	82.50	− 0.63	0.19	− 0.33
Rock	%	3.38	11.78	0.00	48.00	0.77	0.43	− 0.35
Sand	%	7.35	12.12	0.00	50.00	0.76	− 0.31	0.03
Silt	%	89.27	18.80	35.00	100.00	− 0.92	0.05	0.12
Velocity	cm/s	6.12	14.83	0.00	60.00	0.86	0.3	− 0.29
Connectivity	%	41.4	34.9	0	100	0.22	− 0.25	− 0.23

The explained variance of each principal component is shown in parentheses

We used Principal Component Analysis (hereafter PCA) to characterize physical habitat structure and to reduce the number of explanatory variables to a small number of largely independent (orthogonal) environmental gradients (see e.g., Amoros and Bornette 2002; Peres-Neto et al. 2003; Heino et al. 2007; Legendre and Legendre 2012; Czeglédi et al. 2016, 2020; Sinha et al. 2019). The PCA was conducted on the correlation matrix of the recorded physical habitat structure variables, using the function “prcomp” in the package *factoextra* 1.0.7. (Kassambara and Mundt 2017). The values of the variables were square-root arcsin transformed in advance of the PCA (Legendre and Legendre 2012; Luck et al. 2013).

According to the PCA analysis, the first three most influential and interpretable environmental gradients with their eigenvalue over 1 and their explained variance over 10% were as follows (for details see Table 3 and Online Appendix 1). The PC1 axis characterized a gradient where relatively deep water bodies with high velocity, and relatively coarse substrate composition (dominantly sand) occupied one end, while relatively shallow water bodies with dense canopy and/or macrophyte cover, woody debris and fine substrate composition (silt) occupied the other end of the gradient. The PC2 axis represented a gradient where sites with relatively high canopy cover, and trees along the bank were situated on one end, while sites with relatively dense macrophyte cover and herbaceous bank vegetation the other end of the gradient. PC3 was determined mainly by canopy cover, herbaceous bank vegetation, woody debris, sand substrate, and the composition of macrophyte vegetation.

We used the component scores of the water bodies along these most influential first three principal components as explanatory variables in further analyses (see below at variance partitioning).

### Landscape metrics

We measured the percentage cover of selected land cover types from the CORINE Land Cover 2018 database GIS layer (European Environmental Agency 2020, http://www.eea.europa.eu; Mag et al. 2011; Portaccio et al. 2021) in 125, 250 and 500 m buffers around each rectangular study plot (modified after Akasaka et al. 2010; Milder et al. 2010; Yabuhara et al. 2019). The studied land cover types were as follows: agricultural, artificial, forest, natural grassland, transitional woodland-shrub, water body and wetland. We measured the percentage cover of each land cover type in Quantum GIS version 3.4.12-Madeira (QGIS 2022).

### Spatial metrics

We used only positively autocorrelated Moran’s Eigenvector Matrices (MEM) from the geocoordinates of the sites as explanatory variables of spatial structuring (Peres-Neto and Legendre 2010; Sattler et al. 2010; Ferenc et al. 2014). We used the “dbmem” function of the *adespatial* package in R for the calculations (version 0.3–20; Dray et al. 2018).

### Variance partitioning analyses

We conducted redundancy (RDA) and associated variance partitioning analyses (Borcard et al. 1992, 2011, 2018; Legendre and Legendre 2012) to quantify the pure and shared effects of the three predictor variable groups (local scale habitat structure, land cover and spatial positioning) on the structure of aquatic and terrestrial bird communities. We used the Hellinger-transformed relative abundance of taxa and foraging guilds separately in the analyses. Consequently, both the relatively short gradients we obtained using preliminary detrended correspondence analyses and Hellinger transformation of the data justify the applicability of linear ordination, such as RDA (Legendre and Gallagher 2001; Peres-Neto et al. 2006; Legendre and De Cáceres 2013; Lorenzón et al. 2016b; Borcard et al. 2018; Henckel et al. 2019; Anderson et al. 2011) Statistical significance of the unique contributions of the three sets of predictors was tested using the “anova.cca” function with 1000 runs in package *vegan* (version 2.6–4; Oksanen et al. 2017). In advance of variance partitioning, separate forward selection of the physical habitat PCA components, the land cover and spatial variables were computed using a permutation-based test with the “ordistep” function of the package *vegan* with 1000 runs (Rush et al. 2014; Hill et al. 2019). Only variables that significantly (alpha = 0.05) contributed to community variability were retained in the final models (Lorenzón et al. 2016b; Hill et al. 2019; Sultana et al. 2022). All analyses were conducted in the R environment 4.2.2 (R Core Team 2022).

## Results

### Aquatic birds

Throughout the transects, we recorded 778 aquatic birds of 33 species (see Table 1 for the species list and relative abundance data). The mallard (*Anas platyrhynchos*), grey heron (*Ardea cinerea*) and little egret (*Egretta garzetta*) were the three most abundant species and had a relative abundance of 38%, 11% and 8% respectively. The three foraging guilds with the highest relative abundance were dabbling ducks (54%), large wading birds (25%) and small wading birds (11%). For the relative abundance data of aquatic species, the total explained variance was 10%, 14% and 15% for the 500, 250 and 125 m spatial scales, respectively. All three variable groups contributed to the explained variance at the 500 m scale, with both pure and shared components (Table 4a), while at the 250 m scale pure local habitat structure, land cover, spatial and the joint (i.e., spatially structured local habitat structure) components were influential. On the other hand, pure land cover, spatial variables, locally structured land cover—the shared component of local and land cover factors—and the intersection of all three variable groups contributed to the explained variance at the 125 m scale. Considering local habitat structure, only the first principal component contributed to the explained variance at each scale. Land cover types contributed differently at the different scales (Table 5): at the 500 m scale the cover of transitional woodland-shrub areas, at the 250 m scale, the cover of wetlands and at the 125 m scale the cover of forests and wetlands affected taxonomic structure significantly. The importance of land cover increased with decreasing scale. The shared components had only a marginal contribution to the explained variance.

**Table 4:** Results of variance partitioning analyses, which shows the proportions of explained variance of pure local (lo), land cover (la), spatial (sp) variables and their shared components at different landscape scales (500 m, 250 m, 125 m)

Aquatic species relative abundance	Local (%)	lo+la (%)	Land cover (%)	Spatial (%)	lo + sp (%)	lo + la + sp (%)
a)						
500 m	18	9	9	36	9	9
250 m	20	0	33	33	13	0
125 m	0	13	40	33	0	13

Aquatic foraging guild relative abundance	Local (%)	lo + la (%)	Land cover (%)	Spatial (%)	lo + sp (%)	lo + la + sp (%)
b)						
500 m	0	7	0	43	14	21
250 m	7	0	0	57	36	0
125 m	7	0	0	57	36	0

Terrestrial species relative abundance	Land cover (%)	la + sp (%)	Spatial (%)			
c)						
500 m	43	43	14			
250 m	63	38	0			
125 m	67	33	0			

Terrestrial foraging guild relative abundance	Land cover (%)	la+sp (%)	Spatial (%)			
d)						
500 m	48	0	52			
250 m	58	0	42			
125 m	70	0	30

**Table 5:** Determinants of the taxonomic and foraging guild structure of aquatic and terrestrial bird communities. Variables that contributed significantly to each variable group in variance partitioning at different landscape scales (500 m, 250 m, 125 m) are listed. PC1 characterizes the main environmental gradient, while the different MEM vectors represent spatial gradients based on Moran’s Eigenvector Matrices

Response	Variable group	500 m	250 m	125 m
Aquatic species relative abundance	Local scale habitat structure	PC1	PC1	PC1
	Land cover	Transitional woodland-shrub	Wetland	Wetland, forest
	Spatial vector	MEM3	MEM3	MEM3
Aquatic guild relative abundance	Local scale habitat structure	PC1	PC1	PC1
	Land cover	Transitional woodland-shrub	–	–
	Spatial vector	MEM3	MEM3	MEM3
Terrestrial species relative abundance	Local scale habitat structure	–	–	–
	Land cover	Agricultural	Agricultural, transitional woodland-shrub	Agricultural, transitional woodland-shrub
	Spatial vector	MEM8	MEM8	MEM8
Terrestrial guild relative abundance	Local scale habitat structure	–	–	–
	Land cover	Agricultural	Agricultural, natural grassland	Agricultural, natural grassland, transitional woodland-shrub
	Spatial vector	MEM1, MEM5	MEM1, MEM5	MEM1, MEM5

For the relative abundance of aquatic foraging guilds, the total explained variance was 14% at each spatial scale. All three variable groups contributed to the explained variance at the 500 m scale, while land cover did not contribute to the explained variance at other scales (Table 4b). At the 500 m scale the only contributing pure component was the spatial variable group, while at other scales pure local, pure spatial and spatially structured local components were represented. Considering local habitat structure, only the first principal component contributed to the explained variance (Table 5). Land cover types (specifically transitional woodland-shrub) proved to be influential only at the 500 m scale (Table 5). The majority of the explained variance was contributed by the pure spatial component at each scale (57%), while the locally structured spatial component comprised the second highest proportion (36%). The pure local component had only a marginal influence on guild-based structure (Table 4b).

### Terrestrial birds

We observed 1192 individuals of 45 terrestrial bird species (see Table 2 for the species list and relative abundance data). The great tit (*Parus major*), common chaffinch (*Fringilla coelebs*) and Eurasian blackcap (*Sylvia atricapilla*) were the three most abundant species and had a relative abundance of 14%, 9% and 8% respectively. Crown insectivores (25%), ground insectivores (18%) and herbivores (17%) were the three most abundant foraging guilds and were present in 25, 18 and 17% relative abundances. For the relative abundance data of terrestrial species, the total explained variance was 6%, 8% and 9% for the 500, 250 and 125 m buffer zones, respectively. Only land cover and spatial variables contributed to the explained variance (Table 4c). At the 500 m scale, both the two pure components and their shared component contributed to the explained variance, but at other scales, no contribution of the pure spatial component emerged. Land cover types contributed differently to the variance at the different scales (Table 5): at the 500 m scale, only the cover of agricultural surfaces contributed to the explained variance, while at other scales the importance of both agricultural and transitional woodland-shrub surfaces emerged. The importance of the pure land cover variable group increased with decreasing spatial scale (from 43% at the 500 m scale to 67% at the 125 m scale). Parallelly, the contribution of spatially structured landscape component decreased with decreasing scale (from 43 to 33%). The pure spatial component had only marginal contribution, and only at the 500 m scale.

For the relative abundance of foraging guilds, the total explained variance was 20%, 25% and 32% at the 500, 250 and 125 m scales, respectively. Only land cover and spatial variables contributed to the explained variance, and only with their pure components at each scale (Table 4d). Similarly to taxonomic structure, land cover types contributed differently to guild structure at the different spatial scales (Table 5). For example, agricultural fields were important at each scale, but natural grasslands were influential only at the 250 m and 125 m scales. In addition, the contribution of transitional woodland shrub surfaces was significant only at the 125 m scale. The importance of the pure land cover variable group increased with decreasing scale, similarly to taxonomic structure. The pure landscape component contributed significantly to the explained variance at each scale, and its contribution further increased with decreasing scale (from 48% at the 500 scale to 70% at the 125 m scale).

## Discussion

### Total variance

We found low total explained variances for both aquatic and terrestrial bird communities, which varied between 9 and 32%. These values could have been even lower if we had not accounted for detection probability bias in the methodology, such as by conducting three count sessions and alternating the order of visits (Thompson 2002; Cornils et al. 2015). The lowest and highest total explained variances were found for the taxonomic and functional structuring of terrestrial birds, respectively, while aquatic birds showed intermediate variance values. Several factors may contribute to the differences in the predictability of terrestrial and bird communities and between taxonomic and functional approaches, including for example the range of the underlying environmental gradient(s), the number of environmental and spatial predictors structuring the communities, or the number of taxa or functional groups used in the analysis (Heino et al. 2007, 2013).

Low total explained variance values are more general than exceptional in those community ecological studies which deal with the importance of environmental structuring and space using variance partitioning procedures (Sattler et al. 2010; Meffert et al. 2013; Meynard et al. 2013; Heino et al. 2015a). Several authors assumed that low total variance values can be due to nondeterministic factors, such as unmeasured biotic and abiotic variables, or more complex spatial structure compared to what can be characterized by field observations (Borcard et al. 1992; Sattler et al. 2010; Henckel et al. 2019; Ovaskainen et al. 2019). On the other hand, others argued that the interpretation of ‘unexplained variation’ as random variation caused by unmeasured factors is generally inappropriate (e.g., Økland 1999; Meffer and Dziock 2013). Due to the high variability in ecological data, and elusive species environmental relationships, Økland (1999) recommended concentrating on the relative contribution of variation explained by different sets of explanatory variables rather than focusing on the importance of explained and unexplained variations. Stegen and Hurlbert (2011) also argued that low explained variance does not necessarily indicate weak dispersal limitation and environmental filtering and suggested to use relative proportions of partitioned variances to characterize the relative influences of these two mechanisms. Consequently, we focussed on the interpretation of the relative importance of the different variable groups in the discussion below.

### Local scale habitat structure

As hypothesised, local-scale habitat structure of the waterbodies and the riparian zone proved to be important for aquatic birds in the case of both taxonomic and foraging guild structure. However, its contribution as a pure or as a shared component varied depending on spatial scale, presumably due to its interference with land cover variables at different measurement scales. Several papers considered the importance of the habitat structure of water bodies on aquatic bird species composition (Godinho et al. 2010; Arruda Almeida et al. 2016; Lorenzón et al 2016b), but, to the best of our knowledge, this study is the first which used a variance partitioning approach to comparatively examine the importance of different variable groups on foraging guild structure.

The first principal component was the only local scale habitat gradient which significantly influenced aquatic species, including both taxonomic and foraging guild structures. This is not surprising since PC1 characterized the most substantial changes in habitat quality, embracing differences in area, depth, flow velocity, and composition of substrate, aquatic plant and riparian vegetation. Interestingly, hydrologic connectivity correlated only moderately with these habitat variables. This finding shows the complex relationship between local scale habitat structure and hydrological connectivity and also reveals that connectivity in itself cannot substitute other variables for characterizing the habitat structure of floodplain water bodies. These results on the importance of complex local scale habitat gradients correspond with the findings of former studies (e.g., Godinho et al. 2010; Lorenzón et al. 2019; Arruda Almeida et al. 2016; 2018; Fluck et al. 2020).

### Land cover

To the best of our knowledge, no scientific papers aimed to compare the contribution of habitat characteristics of the water bodies and land cover elements in the structuring of terrestrial floodplain bird communities. Land cover proved to be a more important determinant of both the taxonomic and functional structure of terrestrial birds than aquatic ones. This result is not surprising since terrestrial birds use the terrestrial landscape for foraging and nesting, while aquatic species are connected more to the water bodies and wetlands. However, the zero contribution of local scale habitat structure of the waterbodies in the structuring of terrestrial bird communities is somewhat surprising, since floodplain water bodies and wetlands can influence insectivore bird populations by the density of the swarming aquatic invertebrates, known as aquatic subsidies, such as mayflies (Ephemeroptera) and stoneflies (Plecoptera), which can be important foraging sources in early spring, before the later pulse of canopy insects, such as aphids (Hemiptera) and caterpillars (Lepidoptera) (Nakano and Murakami 2001; Murakami and Nakano 2002; Iwata et al. 2003; Schilke et al. 2020; Wesner et al. 2020).

The contribution of land cover variables to the explained variance generally remained stable or increased with decreasing spatial scale of the evaluation area (here from 500 to 125 m). This finding thus supports, at least partly, our hypothesis that bird communities respond relatively strongly to the heterogeneity of land cover, especially at finer spatial scales, which may better fit their territory, foraging- and nesting area (see also Henckel et al. 2019; Meffert and Dziock 2013). According to Henckel et al. (2019), this statement can also stand for individual land cover types, as some types are more characteristic factors for fine-scale territories and feeding grounds of both terrestrial and aquatic species respectively, while others are more influential on larger scales. In our case, for aquatic birds, transitional woodland-shrub surfaces (i.e., shrublands) were only significant at the 500 m scale, while wetlands were crucial at the two smaller scales. For terrestrial birds, agricultural lands were important at each scale, while shrublands and natural grasslands were influential at the 250 and 125 m scales.

In our study, the variance of aquatic bird species structure was influenced by shrublands, wetlands and forests. The cover of shrublands was the only crucial land cover variable in the variance of aquatic bird foraging guild structure. The vertically complex but open habitat structure of shrublands can influence the structure of aquatic bird communities, for instance, numerous species groups, such as large waders prefer to nest in that particular habitat type in floodplain forests (Liang et al. 2007; Parkes et al. 2012). On the other hand, raptors favouring aquatic habitats can hunt their prey with higher success in open areas of shrublands (Davis et al. 2009). Wetlands are crucial for aquatic birds, supplying a variety of microhabitats from wet grasslands, silty beaches, across different associations of aquatic macrophytes to even open water surfaces. Such complex habitats can serve as feeding or nesting grounds for diverse aquatic bird communities (Lorenzón et al. 2016a, b; Galib et al. 2018; Htay et al. 2023). Similarly, forests provide shelter, and nesting microhabitats for ground-, canopy- and cavity-nesting water birds alike (Lemelin et al. 2010; Andrade et al. 2018; Sinha et al. 2022).

Agricultural and shrubland surfaces were generally important drivers in the structuring of terrestrial bird communities (i.e., both for species and foraging guilds), while natural grasslands only influenced the variance of foraging guild structure. The substantially simple habitat structure of monocultural agricultural lands only can harbour poor bird communities, resulting in species and foraging guilds preferring open habitats that can tolerate such a low diversity of foraging sources (Best et al. 1995; Selwood et al. 2015; Socolar and Wilcove 2019). As the vertical structure of shrublands is more complex than open grasslands but less so than closed forests, this particular habitat type can harbour species of open habitats as well as forest edge or open forest-dwelling species. Thus, the cover of this habitat type can substantially influence the explained variance of species structure in a landscape (Knutson 1995; Lorenzón et al. 2016a). The cover of natural grasslands can influence the presence of numerous foraging guilds, since for example shrub insectivores live in either shrublands or forests, while bark-foraging and canopy insectivores prefer forest habitats and hardly can be present in grasslands (Reid et al. 2016; Fourcade et al. 2018; Senner et al. 2021).

### Space

We found a relatively high contribution of purely spatial variables to the explained variance in the case of both aquatic and terrestrial birds and both for taxonomic and functional structure. This suggests that dispersal limitation would be an influential factor in community structuring (Gianuca et al. 2013; Henckel et al. 2019). On the other hand, the decreasing contribution towards the smaller scales suggests the decreasing importance of dispersal limitations (Henckel et al. 2019). This result was more expressed in terrestrial birds, which in our case are mainly territorial forest species, showing only post-natal dispersion in the forest corridors on relatively small scales (Machtans et al. 1996; Laurance and Gomez 2005; Seaman and Schulze 2010). On the other hand, most aquatic birds are considered large distance dispersers, regarding their movements between foraging habitats (Reynolds et al. 2015; Coughlan et al. 2017). Henckel et al. (2019) suggested that pure spatial structuring may be explained by individual movements during the breeding season rather than dispersal limitation sensu stricto. Purely spatial variables may also indicate the random (i.e., environmentally independent) aggregation of some species and/or functional groups during their movement across the landscape by mass-effect mechanisms (Meynard and Quinn 2008; Watson and Watson 2015; de Souza Leite et al. 2022). In this regard, mass-effects may increase in importance with decreasing distances between sites and small spatial extent surveyed (Heino et al. 2015b).

### Joint portions

The shared component of space with local or land cover variables proved to be also important, especially in the functional structuring of aquatic birds (here spatially structured local scale habitat structure), and in the taxonomic structure of terrestrial birds (spatially structured land cover). Spatially structured environmental components (both local habitat structure and landscape features alike) indicate the spatial distribution of important environmental gradients that influence the dispersion of bird species and foraging guilds (Sattler et al. 2010). For example, for aquatic birds, this component embraces the sorting of species and/or functions along the lateral habitat and connectivity gradients from the main river to the most secluded backwaters (see also Parkinson et al. 2002). For terrestrial species, the spatially structured land cover component mirrors the effect of spatial heterogeneity in the distribution of land cover types (e.g., shrubland surfaces) both longitudinally along the river and laterally along the floodplain (Renöfält et al. 2005). Nevertheless, the large variability in the contribution of pure and shared variance components between measurement scales suggests that the effect of environmental heterogeneity, space and neutral or stochastic mechanisms cannot be easily dissected in the case of floodplain bird communities, similarly to other ecosystems or organism groups (see e.g., Borcard et al. 1992; Sattler et al. 2010; Stegen and Hurlbert 2011).

## Conclusions

In conclusion, the structuring of floodplain bird communities showed high context-dependency, similar to many other studies on the metacommunity structuring of ecological communities. Generally, local scale characteristics of the waterbodies and the riparian zone proved to be less influential in community structuring than land cover and spatial variables both for aquatic and terrestrial birds and both for taxonomic and foraging guild structure. The importance of purely spatial variables suggests that mass-effect mechanisms also shape the structuring of floodplain bird communities, besides species sorting mechanisms. Mass-effect may have contributed to the low predictability of community structuring, despite the use of a variety of environmental variables. The predictability of community structuring was also influenced by the measurement scale of land cover variables (i.e., 500, 250 or 125 m radius around the survey transect) and was generally highest at the lowest spatial extent. Overall, these results indicate the relatively strong response of floodplain bird communities to heterogeneities in land use, but also suggest that dispersal dynamics of birds across the floodplain is also critically important to understand the structuring of bird communities, which should be considered by conservation management.

## Supplementary Information

Below is the link to the electronic supplementary material.

Appendix 1: The ordination plot of the PCA analysis of the local scale habitat variables. Supplementary file1 (PDF 7 KB)

## Data Availability

The data presented in this study are available on request from the corresponding author.
