# Supplementary figures and images for: Drivers of the taxonomic and functional structuring of aquatic and terrestrial floodplain bird communities
Source: Landsc Ecol. 2024 Sep 4;39(9):174. doi: 10.1007/s10980-024-01948-3 (PMC11374819; doi:10.1007/s10980-024-01948-3)

PCA – Biplot

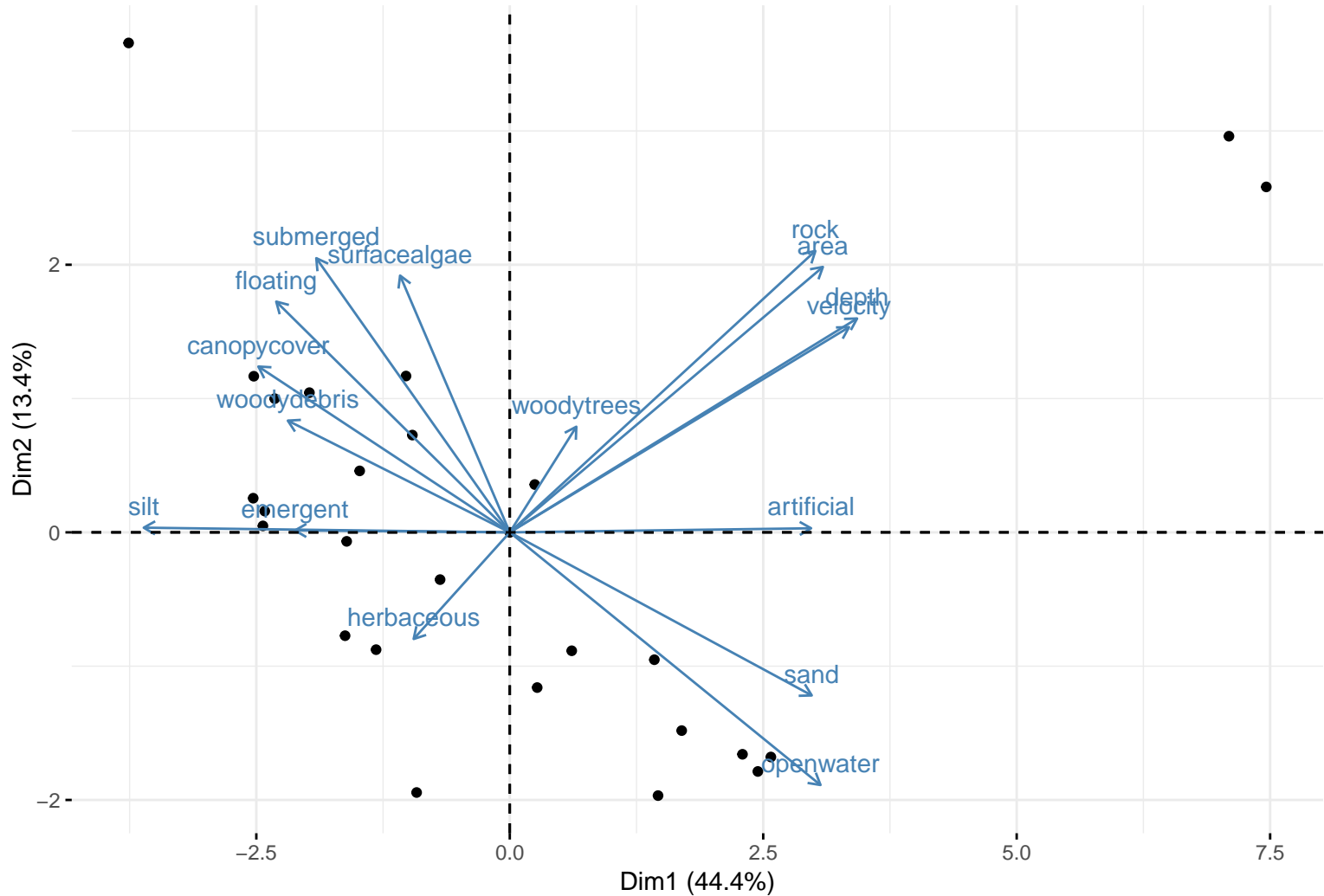

Supplement: Supplementary file 1 — Appendix 1: The ordination plot of the PCA analysis of the local scale habitat variables. Supplementary file1 (PDF 7 KB) [file 10980_2024_1948_MOESM1_ESM.pdf]
